# The reduced genome of a heritable symbiont from an ectoparasitic feather feeding louse

**DOI:** 10.1186/s12862-021-01840-7

**Published:** 2021-06-02

**Authors:** Leila Alickovic, Kevin P. Johnson, Bret M. Boyd

**Affiliations:** 1 Center for the Study of Biological Complexity, Virginia Commonwealth University, 1000 W. Cary St., Suite 111, Richmond, VA 23284-2030 USA; 2 Illinois Natural History Survey, Prairie Research Institute, University of Illinois, Champaign, IL USA

**Keywords:** Phthiraptera, Endosymbiont, Keratin, Genome reduction, Metabolic complementation

## Abstract

**Background:**

Feather feeding lice are abundant and diverse ectoparasites that complete their entire life cycle on an avian host. The principal or sole source of nutrition for these lice is feathers. Feathers appear to lack four amino acids that the lice would require to complete development and reproduce. Several insect groups have acquired heritable and intracellular bacteria that can synthesize metabolites absent in an insect’s diet, allowing insects to feed exclusively on nutrient-poor resources. Multiple species of feather feeding lice have been shown to harbor heritable and intracellular bacteria. We expected that these bacteria augment the louse’s diet with amino acids and facilitated the evolution of these diverse and specialized parasites. Heritable symbionts of insects often have small genomes that contain a minimal set of genes needed to maintain essential cell functions and synthesize metabolites absent in the host insect’s diet. Therefore, we expected the genome of a bacterial endosymbiont in feather lice would be small, but encode pathways for biosynthesis of amino acids.

**Results:**

We sequenced the genome of a bacterial symbiont from a feather feeding louse (*Columbicola wolffhuegeli*) that parasitizes the Pied Imperial Pigeon (*Ducula bicolor*) and used its genome to predict metabolism of amino acids based on the presence or absence of genes. We found that this bacterial symbiont has a small genome, similar to the genomes of heritable symbionts described in other insect groups. However, we failed to identify many of the genes that we expected would support metabolism of amino acids in the symbiont genome. We also evaluated other gene pathways and features of the highly reduced genome of this symbiotic bacterium.

**Conclusions:**

Based on the data collected in this study, it does not appear that this bacterial symbiont can synthesize amino acids needed to complement the diet of a feather feeding louse. Our results raise additional questions about the biology of feather chewing lice and the roles of symbiotic bacteria in evolution of diverse avian parasites.

## Background

Insects require an exogenous source of macro- and micronutrients to complete development and reproduce [1–3]. These nutrients are obtained from the insect’s diet [1–3] or from microbes that reside within the insect, known as host-beneficial endosymbionts [4, 5]. Some insects that parasitize plants and vertebrate animals are dependent on microbes for nutrients [6–8]. This is because parasites often possess specialized morphology that allows them to exploit host resources; however, these morphological adaptations prevent the parasites from utilizing other resources. While a host resource may be rich in many nutrients, it may lack one or a few nutrients that are essential for insect development and beneficial microbes can provide these missing nutrients [6, 8].

Genome sequencing has advanced investigations of insect-microbial symbioses [5]. Up to a third of insect species are host to beneficial microbes [4, 9]. Many beneficial microbes identified within insects are bacteria [10], and these bacteria often have small genomes, when compared to free-living and pathogenic bacteria [5]. These small genomes contain the genes needed to complete basic bacterial cellular functions, but also the metabolic pathways needed to provide the host with nutrients absent in the host’s diet [7, 11]. While identifying the genes underlying metabolism in the genome of a symbiotic bacteria would not prove the bacteria are supplying the host with metabolites, it is a first step in identifying a candidate role for these bacteria. Therefore, we can make predictions and provide an initial test of hypotheses using genome sequencing.

There are approximately 500 species of blood feeding sucking lice (Insecta; Phthiraptera; Anoplura), while there are over 4500 species of chewing lice (Insecta; Phthiraptera; Ischnocera and Amblycera) [12, 13]. Extensive research has elucidated the roles and history of bacterial endosymbionts of blood sucking lice [6, 14–19], but much less is known about the closely related chewing lice. Chewing lice either feed on blood by chewing through a host’s skin [20] or consume dermal elements, such as feathers or sebaceous secretions [13]. Most species of chewing lice in the diverse suborder Ischnocera parasitize birds and nearly exclusively consume feathers [13]. Just like blood feeding lice, symbiotic bacteria have been identified in multiple species of feather chewing lice (Phthiraptera: Ischnocera: *Columbicola*) that parasitize dove and pigeon species (hereafter doves) [21, 22]. Therefore, dove lice provided us with a system to study microbial symbioses in feather chewing lice.

Feather feeding lice primarily consume the barbs of downy feathers. Dove lice specifically ingest feathers barbs from feathers found on the abdominal region of birds [23–26] and adult lice can persist in off the body of the host when provided with appropriate abdominal feathers [27]. Feather barbs are composed of keratin, and analysis of feather keratin from chickens would suggest feather keratin lacks four of the 10 amino acids insects require in their diet (histidine, lysine, methionine, and tryptophan) [28, 29]. Given that feather chewing lice are known to harbor endosymbiotic bacteria, we hypothesized that they might provide the lice with a source of these four amino acids. In this study we did two things to address this hypothesis. (1) We predicted amino acid composition of feather keratin from a dove species. (2) We used genome sequencing and annotation to determine if a symbiotic bacterium from a feather chewing louse (parasitizing a dove) retained genes required for amino acid metabolism, specifically for metabolisms of histidine, lysine, methionine, and tryptophan.

We predicted the amino acid content of feather keratin in the rock dove (Aves: Columbifomes: *Columba livia*) by identifying feather keratin encoding genes in the genome of the rock dove and determining the predicted contribution of each amino acid to the primary structure of keratin. We initially considered sequencing and annotating the genome of the beneficial endosymbiont from lice parasitizing the rock dove [21, 22]. However, we were concerned that this symbiont’s genome may not be acceptable for testing our hypothesis. Given a few exceptions, endosymbiotic bacteria that possess a small genome also show a bias in nucleotide base composition, with adenine–thymine (AT) bases representing the majority of bases [30]. Fukatsu et al. [21] and Smith et al. [22] reported sequences for the symbiotic bacteria isolated from lice parasitizing the rock dove. These sequences were composed of more than 50% guanine-cytosine base pairs (*fusA*, 51% GC; *groEL* 53% GC). In comparison, the orthologous sequences obtained from the highly reduced genome of the endosymbiont of the human head louse (*Candidatus* Riesia pediculicola) contain a majority of AT base pairs (*fusA,* 38% GC; *groEL,* 35% GC). While insect beneficial endosymbionts have small genomes, they are believed to have been derived from bacterial with larger genome and that their genomes are reduced after the symbiotic relationship is formed [5, 31–33]. A majority GC composition could be associated with a larger genome that has not been reduced and would be poor choice for our study.

During a recent study of pigeon and dove lice [34], we identified a partial genome assembly from an endosymbiont that was enriched for AT base pairs and appeared well suited for our study. Therefore, we chose to focus on this louse and its endosymbiont with the assumption that the genome would be small and well suited for our study. Here we present the genome of a symbiotic bacteria identified from an ischnoceran feather chewing louse *Columbicola wolffhuegeli* (Phthiraptera: Ischnocera). This louse parasitizes the Pied Imperial Pigeon (*Ducula bicolor*; Aves Columbidae) in Australia.

## Results

To understand the composition of dove feather barbs, we used the genome of the rock dove to predict the amino acid composition of feather keratin. We evaluated our predictive process by making the same predictions of amino acid composition of feather keratins in the chicken (*Gallus gallus*) and then compared the results to known feather keratin amino acid composition as determined by Arai et al. [29] from feather barbs. Our analysis predicted amino acid composition for feather keratin that was very similar to that reported by Arai et al. [29] and amino acid composition was similar between rock doves and chickens (Table 1). We found, in agreement with Arai et al. [29], that histidine, lysine, methionine, and tryptophan are rare in, or absent from, feather keratin of the chicken and rock dove.

**Table 1:** Predicted amino acid content of feather keratin from *Gallus gallus* (chicken) and *Columba livia* (rock dove) as compared to determined amino acid content for chicken [29]

Amino	*Columba livia*	*Gallus gallus*	Chicken
Acid	(Predicted)	(Predicted)	(Arai et al. 1983)
Phenylalanine	2.60%	3%	3.90%
Histidine	0.70%	0.60%	0%
Isoleucine	4.60%	4.50%	4.60%
Lysine	0.30%	0.20%	0%
Leucine	6.30%	6.60%	7%
Methionine	1.20%	1.20%	0%
Arginine	3.50%	4.60%	4.50%
Threonine	5.10%	5.10%	4.70%
Valine	8.90%	7.90%	7.40%
Tryptophan	0.30%	0.20%	0%

Values are presented as a percent of overall amino acid content (contribution of amino acids that insect do not require from an exogenous source are not shown)

**Fig. 1:**
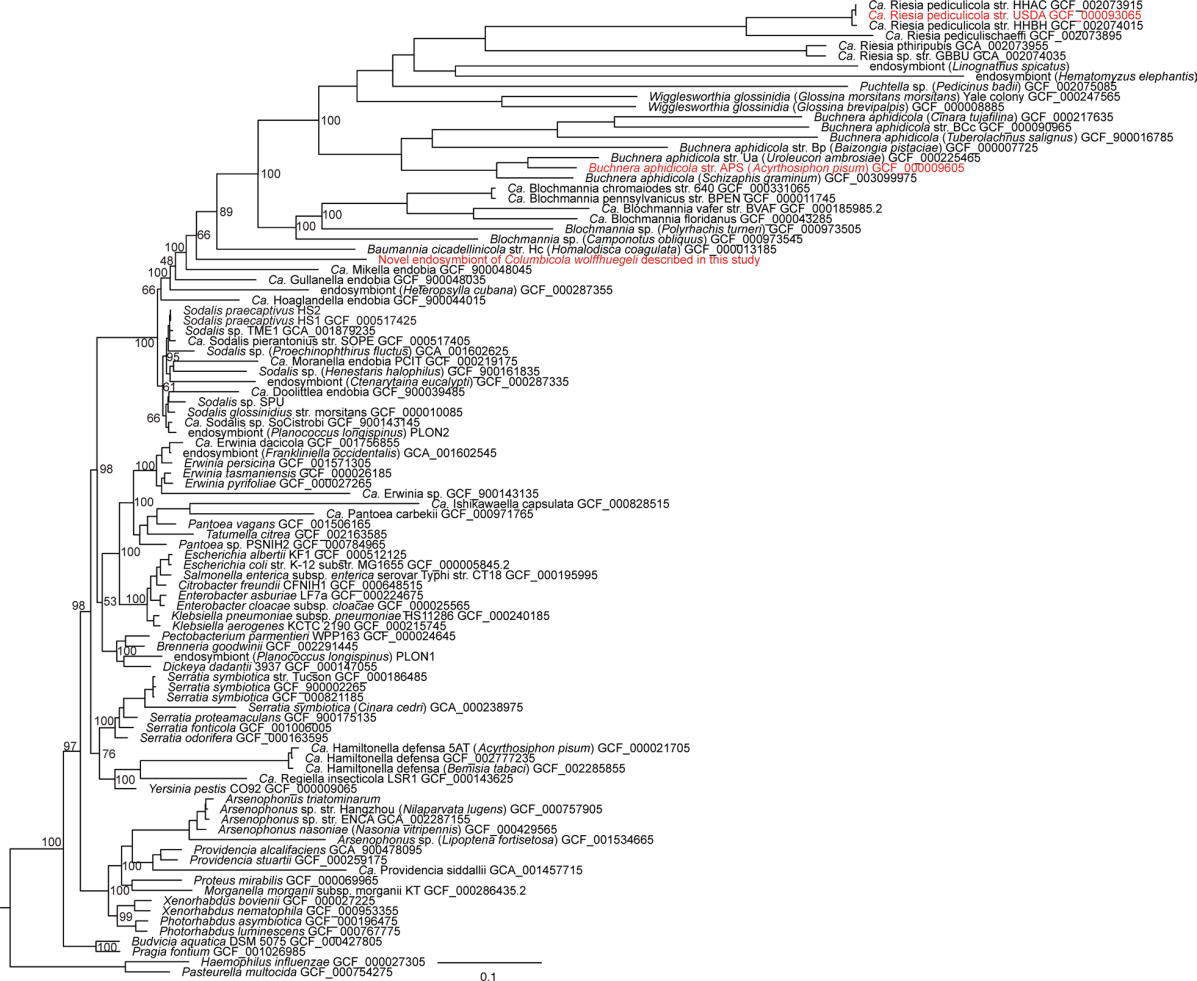
Phylogenetic relationship of the *Columbicola wolffhuegeli* endosymbiont to other γ-proteobacteria. Tree is based on nhPhyML search that started from our initial maximum-likelihood tree obtained from a RAxML search. Values at nodes represent selected bootstrap values obtained from the initial RAxML starting tree. Red taxon labels represent the *C. wolffhuegeli* endosymbiont described in this study and the two taxa that were used in our comparative genomics study. *Ca*. *Candidatus*

Assembly of the *C. wolffhuegeli* endosymbiont genome yielded two contigs totaling 797,418 bp in length (contig lengths were 594,011 and 203,407 bp in length) with 30% of bases being a G or C and was predicted to encode 612 protein-coding genes. The assembly was determined to be near complete (94.58% complete) and no contamination was detected using CheckM [35]. The 16S rRNA sequence within the genome assembly was consistent with previously described endosymbionts from *Columbicola* species [21, 22]. Phylogenomic analysis placed the novel *C. wolffhuegeli* endosymbiont within a clade dominated by endosymbionts (Fig. 1) and *Sodalis* species. Monophyly of this clade was supported by both bootstrap replicates and through comparing alternative phylogenetic arrangements.

**Fig. 2:**
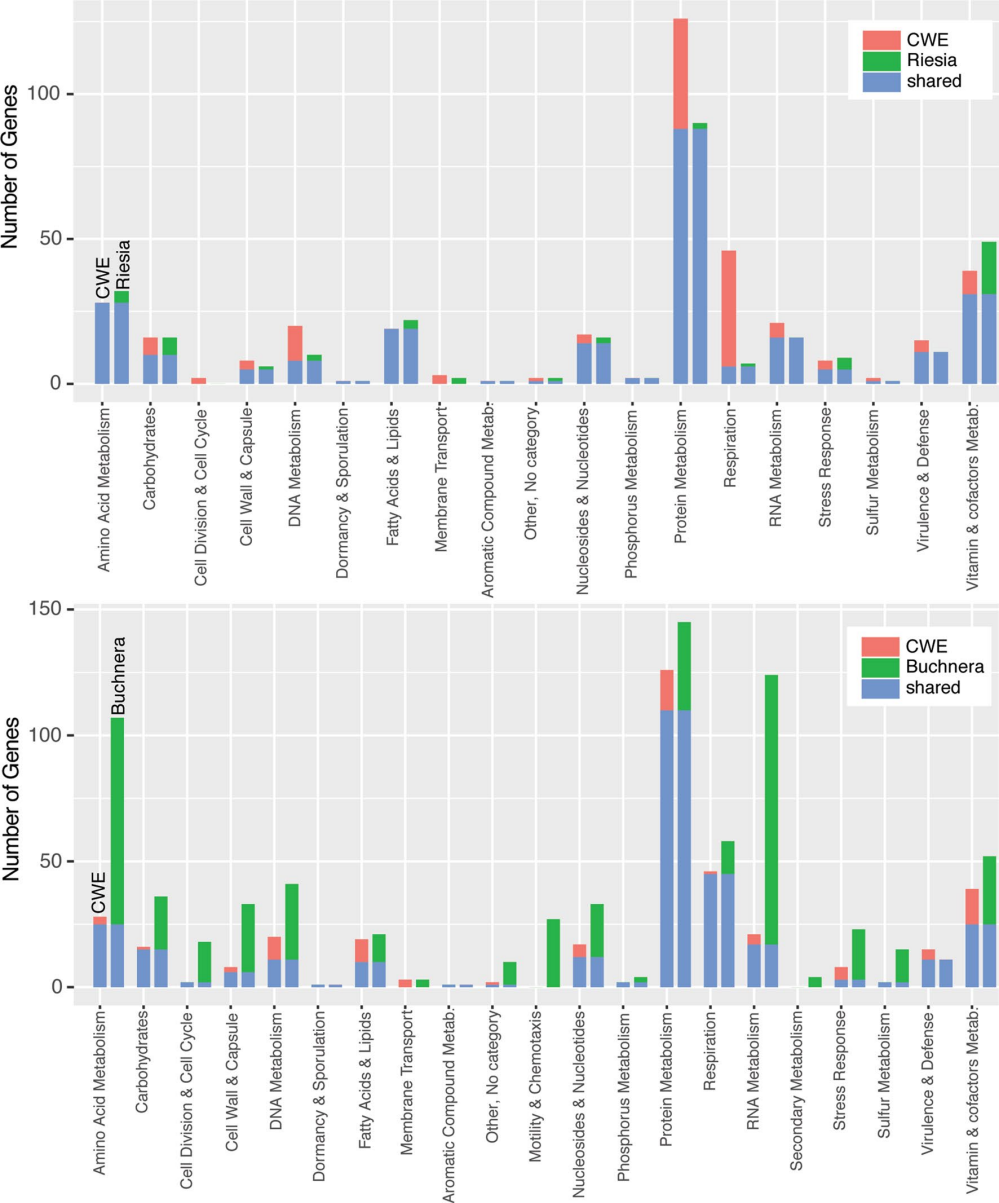
Amino acid and B-vitamin predicted biosynthensis pathways in the *Columbicola wolffhuegeli* endosymbiont (CWE) genome, blood sucking louse endosymbiont *Candidatus* Riesia pediculicola USDA (*Riesia*) genome and phloem feeding insect endosymbiont *Buchnera aphidicola* APS (*Buchnera*) genome. Solid boxes indicate pathways is predicted to be intact, gradient boxes indicate the pathway is incomplete, and empty boxes indicate the pathway is absent

Suspecting the diet of *C. wolffhuegeli* may lack four amino acids, we identified genes encoding enzymes involved in the metabolism of amino acids in the *C. wolffhuegeli* endosymbiont genome through gene discovery and comparisons to two insect endosymbionts *Buchnera aphidicola* and *Ca*. Rieisa pediculicola (Fig. 2). We found that genes expected to underlie metabolism of amino acids are nearly absent in the *C. wolffhuegeli* endosymbiont, when compared to *B. aphidicola* (Fig. 2; analysis guided by results of [7]). Specifically, pathways for synthesis of histidine, methionine, and tryptophan were absent, but the biosynthetic pathway for lysine was predicted to be largely complete (missing only one gene). We attempted to identify other functions of this symbiont. The closely related human blood feeding louse endosymbiont, *Ca.* Riesia pediculicola, is known to supply its host with B-vitamins that are absent in the louse’s diet of blood [6]. Therefore, we attempted to identify genes underling metabolism of B-vitamins in the *C. wolffhuegeli* endosymbiont. We found that metabolism of B-vitamins in *C. wolffhuegeli* endosymbiont is predicted to be reduced in comparison to the human blood feeding louse endosymbiont, *Ca.* Riesia pediculicola (Fig. 2). Specifically, we failed to find all of the genes that underlie biosynthesis of folate (vitamin B-9) in the *C. wolffhuegeli* endosymbiont genome, which are present in the *Ca.* Riesia pediculicola genome (following analysis of [11, 19]). We identified the genes *panB* and *panC*, but not *panE* in the *C. wolffhuegeli* endosymbiont genome*,* all three of which are involved with pantothenic acid biosynthesis (vitamin B-5) in *Ca*. Riesia pediculicola. We also failed to find genes *coaABCD* involved in Co-enzyme A synthesis from pantothenic acid. Metabolism of biotin and riboflavin was largely conserved in both species.

**Fig. 3:**
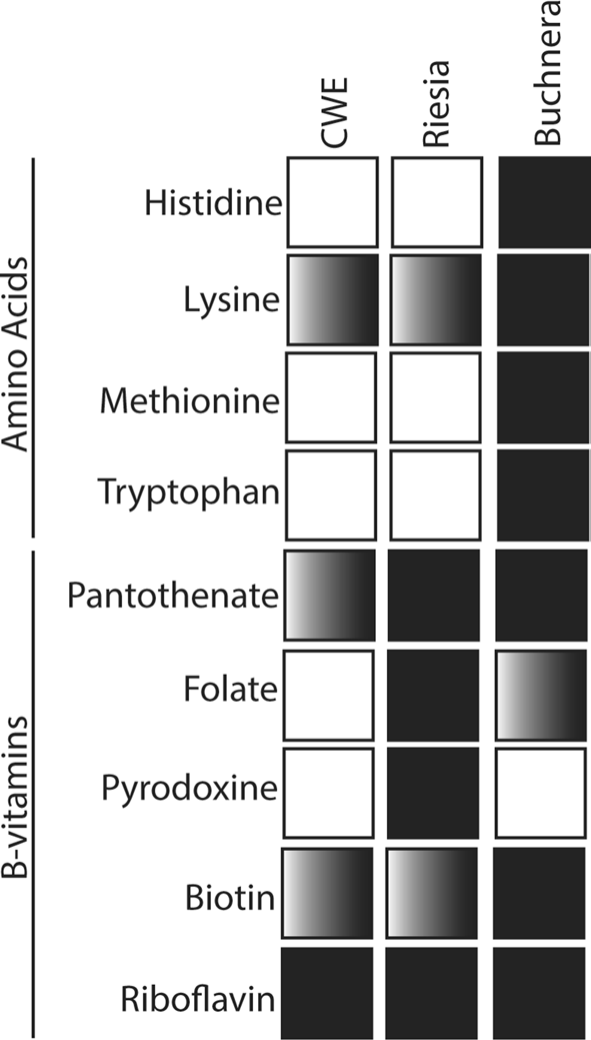
Comparison of total genes by functional category between *Columbicola wolffhuegeli* endosymbiont and two other endosymbionts based on metabolic predictions. (Top) Comparison of *C. wolffhuegeli* endosymbiont and an endosymbiont from a blood feeding louse (*Candidatus* Riesia pediculicola). (Bottom) Comparison of *C. wolffhuegeli* endosymbiont and an endosymbiont from a phloem feeding insect (*Buchera aphidicola*). Red and green bars indicate private functions and blue bars indicate functions found in both genomes being compared. *Metab.* Metabolism, *CWE* *Columbicola wolffhuegeli* endosymbiont

Beyond specific comparisons of amino acid and B-vitamin metabolism, gene content in the *C. wolffhuegeli* endosymbiont genome was more similar to the human blood feeding louse endosymbiont genome than to *B. aphidicola* (Fig. 3). Exceptions to this trend included protein and energy metabolism, in which the *C. wolffhuegeli* endosymbiont is more similar to *B. aphidicola. Ca.* Riesia pediculicola appears to lack some ribosomal proteins and ATP synthase [11], while *B. aphidicola* and the *C. wolffhuegeli* endosymbiont appear to retain the genes encoding the proteins in each subunit of this ATP synthase.

## Discussion

The acquisition of microbial symbionts allowed insects to acquire novel traits and adopt new ecological niches [36]. Novel trait acquisition through symbiotic bacterial partners appears to have been important in the evolution of obligate parasitism by insects [10], including the evolution of blood feeding by parasitic lice [6, 37]. We hypothesized that microbial symbionts allowed lice to become specialist consumers of feathers by providing the lice with amino acids absent in the downy feather barbules that these lice consume. Our results suggested that these feather barbs are a good source of some amino acids, but a poor source of four amino acids insects require from an exogenous source. However, the genome of the louse’s symbiotic bacterial partner lacks the genes that would underly metabolism three of these four amino acids. We then speculated that B-vitamin synthesis might be important, as it is in blood-feeding lice. While we did find that the *C. wolffhuegeli* endosymbiont genome contains gene underlying some B-vitamins, we failed to support metabolism of pantothenate and folate. These two vitamins are required for the development of blood feeding lice and are obtained from their symbiotic bacteria [6].

**Fig. 4:**
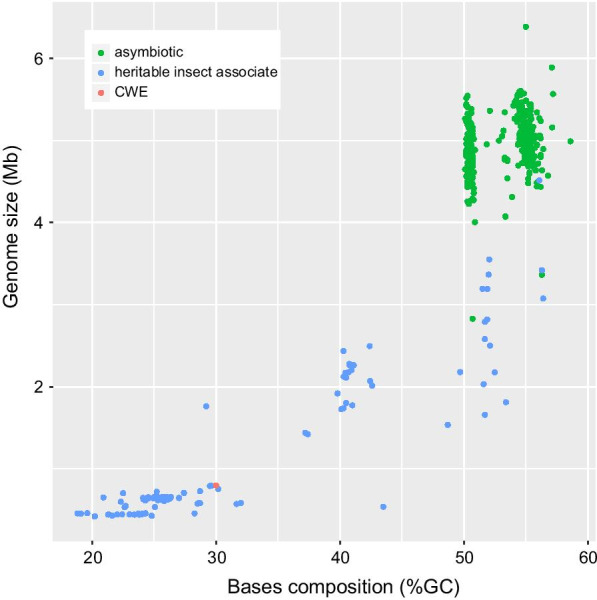
Comparison of γ-proteobacteria genomes by size and base composition. Colors red and green indicate if the genome was derived from an insect endosymbiont or some other type of bacteria. Red indicates the genome of the *Columbicola wolffhuegeli* endosymbiont (CWE)

Many insect-bacterial associations persist for millions of years and the bacteria in these ancient relationships have small genomes when compared to a-symbiotic species (Fig. 4). For example, the genome of the blood feeding human louse endosymbiont, *Ca*. Riesia pediculicola is small, 582,124 bp, and 29% of bases being a GC, considerably small than many free-living or enteric bacterial species in the same taxonomic order (e.g., *Escherichia coli,* 4,641,650 bp 51% GC or *Sodalis praecaptivus,* 5,159,420 bp and 57% GC). The human louse endosymbiont is predicted to have been associated with lice for 12.5–25 mya [11, 19]. Despite lacking genes that would have supported metabolism of amino acids and B-vitamins, the small size and AT content of the genome of the *C. wolffhuegeli* endosymbiont when compared to free-living Enterobacterales, suggests the symbiosis between *C. wolffhuegeli* and its bacterial symbiont may have existed over a long evolutionary time frame.

Overall, the gene content of the *C. wolffhuegeli* endosymbiont genome was most similar to the gene content of the *Ca*. Riesia pediculicola genome, but we did note some differences. Based on our phylogenetic tree, the human head louse and *C. wolffhuegeli* acquired their endosymbionts independently. Therefore, each was derived from a different progenitor and each has evolved independently within its host insect resulting in different outcomes of genome evolution. Because of this, we see differences in gene retention underlying energy production, where the *C. wolffhuegeli* endosymbiont posses these genes, but these genes are absent in *Ca*. Riesia pediculicola, which appears to be dependent on its host for ATP [11]. We also find differences in the predicted modifications of tRNA in these bacteria. Despite these differences, we did observe that gene counts by large functional categories are often closer in the *C. wolffhuegeli* endosymbiont and *Ca*. Riesia pediculicola, than between the *C. wolffhuegeli* endosymbiont and *Buchnera*.

## Conclusions

Our result could suggest that this feather feeding louse gains amino acids from a source other than the endosymbiont. If the endosymbiont is not the source, (1) these amino acids could be derived from microbiota localized in the louse midgut, (2) lice might consume sufficient quantities of feather keratin to obtain sufficient concentrations of rare amino acids, or (3) the lice are consuming some other host resource, in addition to feathers. The latter option would be surprising given that adult lice can live off the host with feathers constituting the only nutritional resource. Also, keratin is resistant to anabolism, but bacterial and fungal isolates breakdown keratin using a series of proteases (reviewed by [38]). There are feather attacking microorganisms present on wild birds [39]. If these microbes can colonize the louse midgut, they may aid in anabolism of keratin and metabolism of amino acids. These communities could be revealed through studies of the louse midgut microbial community. Alternatively, we may have failed to find the relevant genes through assembly or gene discovery. We attempted to control for this problem by ensuring a complete assembly and by comparing our newly sequenced genome to previously described genomes from endosymbionts.

Despite the reduction of genomic space devoted to B-vitamin synthesis in the *C. wolffhuegeli* endosymbiont and the human blood feeding louse endosymbiont have arrived at similar, not identical, gene content. Our phylogenetic tree demonstrates that this is due to convergent evolutionary processes and not due to shared ancestry. Therefore, lice may place similar constraints on genome evolution in heritable endosymbiotic bacteria, regardless of their dietary preferences.

## Methods

### Sample collection and sequencing

A single louse was selected for genomic DNA (gDNA) extraction and whole genome sequencing. Endosymbionts reside within the cells of the louse, so we had to use total gDNA from a whole louse to obtain gDNA from the endosymbiont. The louse we selected for sequencing was collected from *Ducula bicolor,* in Queensland, Australia. Total genomic DNA was extracted from the whole louse specimen using the Qiagen micro kit (Qiagen). The extraction procedure varied from the manufactures recommendation and followed the method described by Boyd et al. [34]. The whole gDNA was used to prepare a sequencing library using the Hyper Library construction kit from Kapa Biosystems (Roche). The library was quantified using qPCR and sequenced on lane for 151 cycles, from both ends of the fragment using the Illumina NovaSeq 6000 platform. The sample was barcoded so that it could be run on the same lane as other samples not described in this study. The fastq files were generated with bcl2fastq v2.20 software package (Illumina).

### Genome composition

Random shotgun sequencing of gDNA from a louse should result in a pool of reads that were derived from the louse genome, the endosymbiont genome, and contaminate gDNA. However, we wanted to assemble the endosymbiont genome in isolation. To obtain an assembly that represents only the endosymbiont genome, we followed an iterative process of genome assembly and isolation developed by Boyd et al. [17]. This method has been successfully employed to sequence and assemble the genomes of other louse endosymbionts [17, 18, 34]. First, we assessed the quality of the read library using FastQC v0.11.9 (Babraham Bioinformatics). Based on the FastQC report, we hard trimmed 5 bases off both the 5′ and 3′ ends of each read using Trimmomatic v0.32 [40]. Next, we created two draft genome assemblies that utilized all of the sequence reads, using two different de novo assembly packages, ABySS v2.1.5 [41] and metaSPAdes v3.14.0 [42]. In both assemblies, we used a k-mer size of 85, as it was found to be optimal by KmerGenie v1.7051 [43]. Once the assemblies were complete, we had to determine which contigs represented portions of louse genome, microbial genome, or contaminate gDNA. To do this we compared all of the contigs, derived from both assemblies, to a custom sequence library consisting of the human louse genome and representative proteobacterial genomes that may be closely related to the endosymbiont (including *Sodalis praecaptivus* HS1, *Arsenophonus nasoniae*, *Ca.* Riesia pediculicola USDA, and *Escherchia coli* K-12 MG1655) using BLASTN v2.10.0 + [44]. From this search, we identified contigs that contained significant hits to bacterial genomes. These contigs were isolated from the larger pool of contigs. Of these contigs, we removed any contigs that were constructed of low coverage k-mers as they likely were derived from contaminate gDNA (endosymbiont contigs had an average coverage of 90X, while contaminates ranged from 3 to 25X coverage). These isolated contigs were then merged using Geneious v2019.2.1 to provide a consensus assembly and treated as a first-pass assembly of the endosymbiont genome.

Next, we sought to improve upon this assembly by conducting a de novo assembly of the endosymbiont genome in isolation from the louse genome or other contaminate gDNA. To do this we isolated the original sequencing reads that contributed to our initial endosymbiont genome assembly by mapping the original sequence reads onto the draft endosymbiont genome assembly. We used Bowtie2 v2.2.6 [45] to align reads to the endosymbiont genome assembly (usage end-to-end function with output of aligned concordantly) and output the aligned reads to a fastq file. This allowed us to create a read-library that contained only reads derived from the endosymbiont genome. From this new library, we conducted a final de novo genome assembly, using metaSPAdes with a k-mer size of 41. It was imperative to ensure we had assembled the genome in its entirety. Therefore, we next sought to determine why we obtained two contigs and not one continuous contig. We attempted to assemble the regions at the end of each contig in isolation using the local assembly tool aTRAM v1 [46]. aTRAM uses a process of finding individual reads that match a target, in this case the last 750 bases of each contig end, collecting the mate-pairs of those matching reads, and finally assembling those reads in isolation. In each instance we found aTRAM returned contigs containing part or all of the genomic copy of 16S rRNA (16S rRNA was present in our initial assembly located near the 3′ end of contig 2), 23SrRNA (which was located within contig 1), and tRNAs. As we looked in detail, we found extremely high coverage around the 5′ and 3′ ends of contigs 1 and 2 as well as in ribosomal and transfer RNAs. We suspect that RNA contamination of the gDNA library or duplicate genomic regions containing RNAs contributed to ambiguities in the de bruijn graph assembly approaches implemented by ABySS and SPAdes. Therefore, we believe our assembly is complete outside of tRNA (involved in protein metabolism) and possibly secondary copies of rRNAs. We wanted to ensure that our initial assembly accurately captured the sequence of the 16S rRNA gene, as its need to verify the identity of the endosymbiont. To validate the assembly, we aligned reads from the entire gDNA library to the sequence of 16S rRNA from *S. praecaptivus* using Bowtie2 and generated a consensus sequence from the resulting SAM file using Geneious. Given the close phylogenetic relationship of the two species (the presence of endosymbionts closely related to *S. praecaptivus* has been established for feather lice) [21, 22], we expected this method isolated only those reads coming from the endosymbiont genome. Using this read-mapping approach we generated a consensus sequence of 16S rRNA from the *C. wolffhuegeli* endosymbiont that was identical to the 16S rRNA sequence found in our initial genome assembly.

### Endosymbiont identity

We sought to place our newly discovered endosymbiont in a phylogenetic tree of γ-proteobacteria, specifically a phylogenetic tree published by McCutcheon et al. [5], that was designed to show the phylogenetic diversity of insect-associated γ-proteobacteria. This tree was based on a set of 130 single copy orthologs which were rigorously evaluated. We sought to identify these orthologs in our endosymbiont genome assembly and add those to an alignment of sequences described by McCutcheon et al. [5]. To do this we had to identify candidate orthologs in our genome. Therefore, we began by annotating the endosymbiont genome using RASTtk pipeline (submitted 2020-03-01) [47–49]. From RAST, we downloaded a fasta file that contained the sequence of each gene predicted in the genome. Using a BLASTP search, we found candidate orthologs shared between the McCutcheon et al. [5] ortholog sets (using the *S. paecaptivus* HS1 protein set as a target and the candidate endosymbiont proteins set as the query) and our newly identified endosymbiont. Using these BLASTP results, we added our endosymbiont genes to the McCutcheon et al. [5] original sequence data. Next, we translated the sequences and conducted a global alignment on all ortholog sets using the global alignment tool MUSCLE, utilizing the BLOSUM62 substitution matrix [50]. Aligned sequences were back-translated to nucleotides, while maintaining the gap characters created in the amino acid alignment. This produced a merged dataset of the *C. wolffhuegeli* endosymbiont into the original ortholog set to use for phylogenetic analysis to identify the placement of the novel endosymbiont in the γ-proteobacterial tree.

First, we wanted to build a maximum-likelihood tree using the GTR model of sequence substitution based on simultaneous analysis of all sequence data. We excluded the third codon positions from each set of ortholog alignments. This was done, because there was significant variation in base composition at the third position, with endosymbionts favoring AT bases at this position and non-symbiotic bacteria having equal base ratios or slightly favoring GC. Next, we determined the optimal partition for estimating free parameters of the substitution model for the remaining first and second codon positions. We followed methodology described by Wickett et al. [51] by splitting each gene alignment into two alignments, one with all first codon positions and the other with all second codon positions. We then conducted a full and unpartitioned ML tree search for both alignments using RAxML v8.2.12 [52]. From these searches, we obtained the shape parameter α and rate parameters used to determine the gamma distribution (Γ). We then used the statistical package R v3.6.1 to determine the optimal number of partitions using PCoA and assign each alignment to a partition using K-means clustering. We found the optimal number of partitions was three. Next, we combined the gene/codon alignments into a single combined alignment and the partition was applied to the concatenated alignment (herein supermatrix). Based on this supermatrix, we found identified a candidate optimal tree using RAxML under the GTR + Γ substitution model, with free parameters estimated for each partition. We implemented 500 partitioned bootstrap replicates of this analysis.

Next, we wanted to test placement of the *C. wolffhuegeli* endosymbiont in the tree using a different model of substitution that may be better suited when free-living and endosymbiont bacteria are combined in phylogenetic analysis. When conducting phylogenetic analysis of endosymbionts, we are concerned about error due to long branch attraction, especially of endosymbionts. This is because endosymbionts favor AT bases. Therefore, we used nhPhyML [53], which implements a non-stochastic DNA substitution model described by Tamura [54] that allows DNA base composition to vary away from equilibrium to evaluate the placement of the *C. wolffhuegeli* endosymbiont in γ-proteobacteria. nhPhyML takes a starting tree and improves upon it using permutations of the tree derived from nearest-neighbor interchange (NNI), evaluating each permutation under an ML criterion. NNI functionally limits a search of alternative trees when compared the search parameters found in RAxML; however, it can optimize starting trees and calculate site likelihoods for those locally optimal trees. From these site likelihoods we can compare alternative trees using CONSEL v0.20 [55] allowing us to compare alternative phylogenetic arrangements. We created seven different starting trees to use with nhPhyML, each presenting a different hypothesis of the evolutionary origins of the *C. wolffhuegeli* endosymbiont. The first tree was the best tree found by RAxML, suggesting the *C. wolffhuegeli* endosymbiont belongs in the clade containing many endosymbionts and members of the genus *Sodalis*. In the six other trees, we removed the *C. wolffhuegeli* endosymbiont from the *Sodalis* clade and placed it elsewhere in the tree (a different placement in each tree) using Mesquite v1.0 [56]. Each permutation was designed to place the *C. wolffhuegeli* endosymbiont into a clade known to contain endosymbionts, include *Yersinia* and the allied endosymbionts *Hamiltonella* and *Regiella*, *Serratia* species, *Erwinia* species, *Pantoea* species, *Brenneria* species, and *Arsenophonus* species. nhPhyML was run independently with each of the starting trees and resulting trees were collected. CONSEL was used to rank the trees from best to worst using multiple statistical tests.

### Genome annotation and metabolic predictions

In this stage of the analysis, we wanted to critically evaluate the metabolic pathways predicted by the genome assembly of the *C. wolffhuegeli* endosymbiont. As mentioned, we used the RASTtk pipeline to annotate the genome assembly, which predicted 612 genes present in the genome. Next we sought to annotate the endosymbiont genome based on alignment with the *S praecaptivus* HS1 genome to validate our annotation. We used LASTZ v.1.02.00 [57] as implement in Geneious to align the genomes. We then copied annotations within alignment blocks from *S. praecaptivus* to the endosymbiont genome and exported the predicted genes as a table. The gene sequences were filtered to remove any predicted genes that were 10% or more shorter than its respective homolog in *S. praecaptivus*. We then used an all-by-all comparisons of genes predicted by the LASTZ alignment and the RASTtk pipeline using TBLASTX. The LASTZ alignment method to identify genes, all of which were predicted by the RASTtk pipeline (based on BLAST returns showing 98–100% overlap and 98–100% identity within the overlap). Therefore, we concluded that the RAST annotation was sufficient for gene discovery.

Next, we investigated the metabolic functions predicted by the genome sequence of the *C. wolffhuegeli* endosymbiont and compared these predictions to similar genomic predictions from other bacteria. Functional predictions between the *C. wolffhuegeli* endosymbiont and three other bacteria were complete in the SEED-Viewer, linked to our RAST annotation. It is important to note that these comparisons are based on predicted function, and while the RASTtk pipeline incorporates sequence comparisons as an extension of the ab initio gene discovery, the comparisons we conducted are not solely based on direct sequence-sequence comparison. We compared the *C. wolffhuegeli* endosymbiont predictions to that of the endosymbiont from the blood sucking human louse, *Ca.* Riesia pediculicola USDA. This comparison was conducted to determine how endosymbiont functions differ depending on the host diet of feathers or blood. Next, we compared the *C. wolffhuegeli* endosymbiont predictions with those from *B. aphidicola* APS. This comparison was conducted, because *B. aphidicola* is an endosymbiont found in insect (aphids) that consume phloem, and *B. aphidicola* retains genes underlying amino acids metabolism [7], and we initially hypothesized the *C. wolffhuegeli* endosymbiont supplies its louse host with amino acids. We used exploratory tools in the Seed Viewer to explore our RAST annotation and compare evidence for B-vitamin and amino acid metabolism in detail and used the integrated BLAST function to check for missing genes. We then checked for three genes that were predicted to be missing by both RAST comparison and BLAST tools in the original read library. Finally, genes encoding enzymes involved in tRNA modification were identified in *Ca.* Riesia and the *C. wolffhuegeli* endosymbiont genomes using BLAST, implemented in RAST. Amino acid sequences from *E. coli* K-12 MG1655 served as the reference sequence and were selected based on a list of tRNA modification genes in de Crécy-Lagard and Jaroch [58].

### Amino acid composition

We determined the relative amino acid composition of feather keratins in pigeons. First, we obtained a list of known feather keratins from the chicken genome as described by Greenwold and Sawyer [59]. Using these genes as query sequences, we then identified candidate homologs of feather keratin genes in the rock dove genome (assembly Cliv_1.0) using BLASTP (blastdb built upon predicted and translated coding sequences). We also replicated this search with the chicken genome (assembly GRCg6a). We then took the best hit for each feather keratin in the genome and save these to a fasta file. From these sequences we calculated the relative amino acid compositions across all feather keratins. This was done for both rock dove and chicken. We then compared the predicted amino acid composition for chicken to the amino acid composition determined biochemically by Arai et al. [29] to assess the utility of our approach.

## Data Availability

The datasets generated during the current study are available. Raw sequence data has been deposited in the National Center for Biotechnology SRA database under the accession SRR14589. The genome assembly has been deposited in the National Center for Biotechnology WGS under the accession JAESFE010000000. Phylogenetic trees, sequence alignments, comparative genomic data, detailed descriptions of predicted amino acid and B-vitamin synthesis, and descriptions of software usage have been deposited in FigShare under the https://doi.org/10.6084/m9.figshare.12678479.

## Abbreviations

A: Adenine
ATP: Adenosine triphosphate
*B.*: *Buchnera*
BLAST: Basic local alignment search tool
bp: Base pairs
C: Cytosine
*C.*: *Columbicola*
*Ca*.: *Candidatus*
CWE: *Columbicola* *wolffhuegeli* Endosymbiont
Γ: Gamma
G: Guanine
gDNA: Genomic deoxyribonucleic acid
GTR: General time reversible
ML: Maximum likelihood
mya: Millions of years
NNI: Nearest neighbor interchange
PCoA: Principal coordinates analysis
qPCR: Quantitative polymerase chain reaction
RAST: Rapid annotation subsystem technology
*S.*: *Sodalis*
S rRNA: Short ribosomal ribonucleic acid
T: Thymine
tRNA: Transfer ribonucleic acid
